# Ecological Reassembly of the Milk Microbiome and Its Associated Resistome During the Dry Period in Dairy Cows

**DOI:** 10.3390/vetsci13060559

**Published:** 2026-06-05

**Authors:** Lan Ma, Jing Qu, Xiubo Li, Yiming Liu

**Affiliations:** 1 National Feed Drug Reference Laboratories, Feed Research Institute, Chinese Academy of Agricultural Sciences, Beijing 100081, China; 82101221300@caas.cn (L.M.); 821012430496@caas.cn (J.Q.); lixiubo@caas.cn (X.L.); 2 Key Laboratory of Animal Antimicrobial Resistance Surveillance, Ministry of Agriculture and Rural Affairs, Feed Research Institute, Chinese Academy of Agricultural Sciences, Beijing 100081, China

**Keywords:** intramammary infection, dry cow therapy, milk microbiome, resistome, metagenomic sequencing, dairy cow

## Abstract

The aim of this study was to characterize the coordinated dynamics of the mammary microbiome, antibiotic resistance genes (ARGs), and mobile genetic elements (MGEs) across the dry period, calving, and early lactation. The mammary microbiome undergoes substantial ecological changes across these stages, yet the coordinated dynamics of microbial composition, ARGs, and MGEs remain poorly understood. Here, shotgun metagenomic sequencing was performed on mammary secretion samples collected before dry-off (BM), immediately after calving (ACM), and one month postpartum (AM). The mammary microbiome exhibited a clear “exposure–bottleneck–reassembly” trajectory. BM was characterized by high microbial diversity and the enrichment of environmentally associated taxa, whereas ACM displayed a pronounced immunological bottleneck with markedly reduced microbial diversity and network complexity. During AM, microbial communities partially recovered but remained distinct from the BM state, indicating persistent ecological restructuring after calving. ARGs and MGEs showed parallel dynamics, with broad resistome and mobilome diversity in BM, a sharp contraction in ACM, and a selective re-expansion in AM. Network analysis further revealed maximal ecological complexity in BM, increased ARGs/MGEs connectivity in ACM, and partial stabilization in AM. These findings demonstrate that host physiological transitions, together with dry cow therapy (DCT), drive the coordinated remodeling of the mammary microbiome, resistome, and mobilome across the dry period.

## Simple Summary

Understanding how the mammary microbial community changes across the dry period and early lactation is important for improving udder health and reducing unnecessary antibiotic use in dairy cows. In this study, milk samples were collected at three key stages: before dry-off, immediately after calving, and one month postpartum. Before dry-off, the mammary gland was characterized by high exposure to environmental microbial, resulting in a diverse but unstable microbial community. After calving, colostrum-associated immune factors markedly reduced microbial diversity and abundance, creating a transient ecological bottleneck in the mammary ecosystem. During early lactation, the microbial community gradually reassembled, although it did not fully return to the pre-dry-off state. Antibiotic resistance genes and mobile genetic elements showed similar dynamics, with a sharp reduction after calving followed by partial recovery during lactation. These findings suggest that host physiological changes, together with dry cow therapy, jointly shape the ecological and functional restructuring of the mammary microbiome. This work provides new insights into udder health management and supports more prudent antimicrobial use in dairy production.

## 1. Introduction

Intramammary infection (IMI) is one of the most prevalent and economically significant diseases in dairy production systems worldwide [1,2]. Mastitis not only compromises animal welfare but also imposes a substantial economic burden on dairy farms due to its high incidence and the considerable cost per case [3]. Clinical mastitis can result in direct losses of several hundred dollars, whereas subclinical mastitis, despite lacking obvious clinical signs, contributes to even greater hidden losses through reduced milk yields and elevated somatic cell counts (SCC) [4,5]. Therefore, identifying the key stages for the occurrence and control of mastitis is essential for improving dairy herd health and farm profitability.

The dry period is widely recognized as a critical window in the epidemiology of IMI. During this stage, the mammary gland undergoes profound physiological transitions from the cessation of lactation to parturition and the subsequent resumption of milk production, accompanied by tissue remodeling, alterations in the mammary microenvironment, and fluctuations in local immune status [6,7,8]. Following the cessation of milking, the keratin plug has not yet fully formed, the teat sphincter closure is delayed, and intramammary pressure increases, all of which facilitate the invasion of pathogens into the mammary gland [9,10]. Consequently, the dry period represents not only a high-risk phase for new IMIs but also a key opportunity for implementing effective control strategies.

Dry cow therapy (DCT) is a widely adopted management practice in which long-acting antimicrobial agents are infused into each teat at drying-off to eliminate existing infections and prevent new IMIs during the dry period [11]. Previous studies have demonstrated that quarters not receiving DCT have a significantly higher risk of developing new infections during the dry period and are more likely to harbor IMIs at calving [12]. Although the effectiveness of DCT in disease control is well established, its role as a large-scale antibiotic intervention suggests that it may also exert substantial effects on the mammary microbial ecosystem, effects which remain insufficiently characterized.

Recent studies have increasingly recognized the mammary gland as a dynamic microbial ecosystem. Diverse and dynamic microbial communities inhabit the milk of healthy cows and contribute to maintaining mammary homeostasis [13,14]. Longitudinal studies have shown that the diversity and composition of the milk microbiota change significantly from the dry period to lactation and are closely associated with mammary health status [15,16]. In addition, mammary epithelial cells are capable of sensing microbial stimuli and undergoing transcriptional reprogramming through immune-related signaling pathways, indicating a bidirectional interaction between the host and microbiota [17]. In this context, antibiotic resistance genes (ARGs), as key functional components of microbial communities, have ecological roles that extend beyond clinical antimicrobial resistance [18]. Their distribution and dissemination are shaped by both microbial community structure and environmental selective pressures [19]. The physiological changes during the dry period, together with the antibiotic exposure associated with DCT, are likely to jointly influence the reassembly of both the microbiome and resistome [20]. However, longitudinal studies investigating the coordinated dynamics of microbial communities and ARGs across the dry period in healthy dairy cows remain limited.

Therefore, the present study employed a longitudinal design covering three key physiological stages: before dry-off (BM), immediately after calving (ACM), and one month postpartum (AM). Following the administration of DCT at drying-off, shotgun metagenomic sequencing was used to systematically characterize the composition and dynamics of the mammary microbiome and resistome across these stages. By integrating host physiological changes and the antibiotic intervention, this study aimed to elucidate the patterns of microbial and resistome reassembly in the mammary ecosystem during the dry period, thereby providing insights for optimizing mastitis control strategies and promoting rational antibiotic use in dairy production.

## 2. Materials and Methods

### 2.1. Procedures Involving Animals and Sample Collection

This study was conducted from January to March 2024 at a commercial dairy farm in Beijing, China (39.9° N, 116.4° E), where the temperate monsoon climate during the sampling period featured average temperatures ranging from −7 °C to 16 °C without extreme weather events. Twelve healthy Holstein cows approaching the dry period were randomly selected from a commercial dairy farm in Tongzhou District, Beijing, China (herd size: 1000). All cows enrolled in the trial were multiparous and clinically healthy with no signs of mastitis, and the SCC in each quarter was below 200,000 cells/mL. Cows were milked three times daily at 07:00, 14:00, and 21:00, and sampling was performed during the 14:00 session by hand-stripping directly into sterile tubes rather than using milking equipment due to the small sample volume required. Milk samples were collected at three time points. Before dry cow therapy (BM), milk samples were collected, and immediately after the final milking, each quarter received one intramammary infusion of a long-acting cefquinome product (3 g:0.15 g; batch no. DB2230108; Cefquinome Sulfate Intramammary Infusion for Dry Cows, Hebei Yuanzheng Pharmaceutical Co., Ltd., Shijiazhuang, China), followed by one tube of an internal teat sealant (4 g:2.6 g; batch no. 2007001; Bismuth Subnitrate Intramammary Infusion for Dry Cows, Qilu Animal Health Products Co., Ltd., Jinan, China). All treatments were administered via intramammary infusion immediately prior to dry-off. After calving, colostrum (ACM) was collected, and mature milk (AM) was sampled one month postpartum. No additional antibiotic treatments were administered during the sampling period.

To ensure the uniformity and reproducibility of the study, the right front quarter of each cow was selected as representative for the study. Research personnel wiped the udders with a clean, dry cloth to remove bedding and visible contaminants. Milking personnel then performed standard pre-milking sanitation: 2–3 streams of foremilk per teat were discarded, after which a 0.5% iodine predip solution was applied and allowed 30–60 s contact time, and the teats were dried with a dry cloth towel. Approximately 30 mL of milk was collected from the right front quarter of each cow into a sterile sample vial. The samples were then placed in insulated containers, quickly transported to the laboratory, and stored at −80 °C for subsequent analysis.

### 2.2. DNA Extraction, Library Construction, and Metagenomic Sequencing

Prior to kit-based purification, approximately 1.5 mL of milk was centrifuged, and the pellet was transferred to a bead-beating tube containing 750 µL of Lysis Buffer A and 90 µL of Lysis Buffer B. Samples were homogenized using a FastPrep-24 5G instrument (MP Biomedicals, Santa Ana, CA, USA) at 6 m/s for 40 s, followed by centrifugation at 14,000 rpm for 5 min. A total of 500 µL of the supernatant was mixed with Precipitation Solutions 1 and 2 (100 µL each), vortexed, and centrifuged again. Subsequently, 400 µL of the clarified lysate was transferred to a deep-well plate and processed using the VOLA 96 Automated Nucleic Acid Extractor (Shanghai Biohandler Life Technology Co., Ltd., Shanghai, China) according to the “Fecal DNA” program to obtain the purified DNA. Total genomic DNA was extracted from 36 samples using the Mag-Bind^®^ Soil DNA Kit (Omega Bio-tek, Norcross, GA, USA) according to the manufacturer’s instructions. The concentration and purity of the extracted DNA were determined using a TBS-380 and a NanoDrop2000, respectively. The quality of DNA extracts was checked on 1% agarose gel.

The DNA extracts were fragmented to an average size of about 350 bp using Covaris M220 (Gene Company Limited, Xianggang, China) for paired-end library construction. Paired-end libraries were constructed using the NEXTFLEX Rapid DNA-Seq (Bioo Scientific, Austin, TX, USA). Adapters containing the full complement of sequencing primer hybridization sites were ligated to the blunt ends of the fragments. Paired-end sequencing was performed on an Illumina NovaSeq (Illumina Inc., San Diego, CA, USA) at Majorbio Bio-Pharm Technology Co., Ltd. (Shanghai, China) using the NovaSeq 6000 S4 Reagent Kit v1.5 (Illumina, San Diego, CA, USA) (300 cycles) according to the manufacturer’s instructions. The sequence data associated with this project have been deposited in the NCBI Short Read Archive database (Accession Number: PRJNA1455201).

### 2.3. Sequence Quality Control and Assembly

Raw paired-end Illumina reads were trimmed to remove adapters, and low-quality reads (length < 50 bp or with a quality value < 20) were removed using Fastp (version 0.23.0) [21]. After quality filtering, a total of 3,083,053,718 reads were obtained from the 36 milk samples (mean: 84,431,873 reads). To remove host-derived contamination, the cleaned reads were aligned to the Bos taurus reference genome (ARS-UCD1.2) using BWA (http://bio-bwa.sourceforge.net (accessed on 15 August 2025), version 0.7.9a) [22]. Read pairs in which either mate mapped to the bovine genome were discarded. After host removal, a total of 10,159,328 reads were obtained from the 36 milk samples (mean: 282,204 reads), which were used for downstream analyses.

The high-quality, non-host reads were then assembled de novo using MEGAHIT (https://github.com/voutcn/megahit (accessed on 15 August 2025), version 1.1.2) [23]. Contigs with a length ≥ 300 bp were selected as the final assembly result and used for subsequent gene prediction and annotation (Supplementary Table S1). All sequencing and assembly statistics are summarized in Supplementary Table S1.

### 2.4. Gene Prediction, Taxonomy, and Functional Annotation

Open reading frames (ORFs) were predicted from assembled contigs using Prodigal (https://github.com/hyattpd/Prodigal (accessed on 15 August 2025), version 2.6.3) [24]. ORFs with a length of ≥100 bp were retained and translated into amino acid sequences using EMBOSS (http://emboss.open-bio.org/ (accessed on 15 August 2025), version 6.6.0) [25] with the NCBI translation table. A non-redundant gene catalog was constructed using CD-HIT (http://www.bioinformatics.org/cd-hit/ (accessed on 15 August 2025), version 4.6.1) [26] with thresholds of 90% sequence identity and 90% coverage. The abundance of each gene was determined by aligning high-quality reads to the non-redundant gene catalog using SOAPaligner (,https://github.com/ShujiaHuang/SOAPaligner (accessed on 15 August 2025), version 2.21) [27] with a minimum identity of 95%. Microbial taxonomic profiles were calculated as relative abundances based on the total number of non-host reads.

Representative sequences of the non-redundant gene catalog were annotated using Diamond (https://github.com/bbuchfink/diamond (accessed on 15 August 2025), version 0.8.35) [28] with an e-value cutoff of 1 × 10^−5^ against multiple databases: the NR database for taxonomic assignment, the eggNOG database for Clusters of Orthologous Groups (COG) functional classification, the KEGG database for metabolic pathway annotation, the CARD database for ARGs, and the MGEs90 database for mobile genetic elements (MGEs). To account for gene length and sequencing depth, ARG and MGE abundances were normalized as transcripts per million (TPM).

### 2.5. Statistical Analysis

Statistical analyses were performed using the Majorbio Cloud Platform (www.majorbio.com accessed on 3 June 2026) and R software (version 4.1.0). For alpha diversity, the Shannon index was calculated and compared among groups using the Kruskal–Wallis test, followed by pairwise Wilcoxon rank-sum tests with Benjamini–Hochberg false discovery rate (FDR) correction. Beta diversity was assessed by principal coordinate analysis (PCoA) based on Bray–Curtis distances, and the significance of group separation was evaluated using an analysis of similarities (ANOSIM) with 999 permutations and a permutational multivariate analysis of variance (PERMANOVA/Adonis). Venn diagrams were constructed using the Venn package (version 2.4.3) to illustrate shared and unique taxa across sampling stages. Differential abundance analysis at the genus level was performed using the Kruskal–Wallis test for multiple-group comparisons. Linear discriminant analysis effect size (LEfSe) was applied to identify features (MGEs types) that were differentially enriched among stages, using an LDA score threshold of >3.0 and a *p* < 0.05. Before diversity and network analyses, taxa with a relative abundance < 0.01% or those present in <10% of samples were removed to reduce noise. Batch effects were evaluated using PCoA and PERMANOVA; no significant batch-associated clustering was detected (*p* > 0.05).

For co-occurrence network analysis, only genera with a mean relative abundance of >0.01% in at least one stage were retained. Pairwise Spearman correlation coefficients were calculated using the WGCNA package(version 1.74) in R, and only correlations with |r| ≥ 0.6 and FDR-adjusted *p* < 0.05 were considered significant. Networks were constructed separately for each stage (BM, ACM, and AM) and visualized using Gephi (version 0.10.1). Nodes were classified into three categories: microbial genera, ARGs, and MGEs. Positive/negative edge associations were indicated by red/green lines. Network topological parameters (e.g., number of nodes, edges) were computed using the built-in functions of Gephi.

## 3. Results

### 3.1. Microbial Community Dynamics Across Lactation Stages

Summary statistics for sequencing depth, host-read removal, and metagenomic assembly are provided in Supplementary Table S1. Across all samples, the raw sequencing output ranged from approximately 12.0 to 13.8 Gb per sample, corresponding to 79–89 million raw paired-end reads. After quality trimming, a total of 3.08 billion clean reads were retained across the 36 samples (mean: 84.4 million reads per sample).

Following host-read removal against the Bos taurus reference genome, 10,159,328 reads remained across all samples (mean: 282,204 reads per sample), which were used for downstream taxonomic, resistome, and mobilome profiling. Although host sequence filtering significantly reduced the number of microbial reads due to the inherently low biomass of milk samples, the preserved sequencing depth was sufficient to support gene-level annotation and, to some extent, reveal the diversity of the microorganisms. Assembly metrics were stable across samples, with contig numbers ranging from 134 to 3302 and N50 values between 639 and 860 bp. Predicted ORFs per sample ranged from 74 to 558 (excluding one AM sample with substantially higher ORF counts), and average ORF length remained within 230–330 bp. These metrics indicate that the assembly quality and annotation depth were adequate to support downstream functional profiling.

#### 3.1.1. Compositional and Diversity Profiles of Microbial Communities

To investigate the ecological succession of the mammary microbiome across the dry period and early lactation, we compared the genus-level microbial composition among the BM, ACM, and AM samples. Distinct stage-dependent shifts in microbial structure were observed, indicating a profound ecological restructuring of the mammary ecosystem during the host physiological transition.

**Figure 1:**
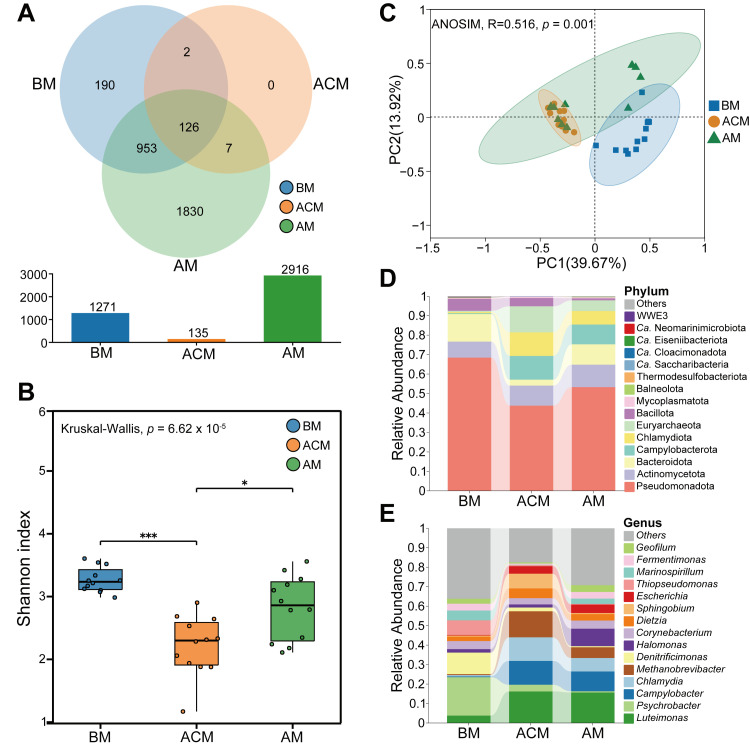
Microbial community structure and taxonomic composition across BM, ACM, and AM. (**A**) The number of shared and unique species among BM, ACM, and AM samples. (**B**) Shannon’s index of alpha diversity. Asterisks indicate significance levels: *p < 0.05, ***p < 0.001. (**C**) Principal coordinate analysis (PCoA) based on Bray–Curtis distances. Each point represents one sample, and shaded ellipses indicate 95% confidence intervals for each group. Blue squares (BM), orange circles (ACM), and green triangles (AM) denote different sample groups. (**D**) Relative abundance of major bacterial phyla across the three stages. (**E**) Relative abundance of major bacterial genera across the three stages.

A total of 190 unique species were identified in BM, whereas AM contained the greatest number of stage-specific species (1830), suggesting that microbial reassembly after calving generated a newly differentiated community rather than restoring the pre-dry-off microbiota (Figure 1A). Only 126 species were shared across all three stages, while BM and ACM shared only two species, highlighting the sharp ecological discontinuity induced during the colostrum stage. Although BM and AM shared 953 species, subsequent diversity and ordination analyses demonstrated that the AM communities remained compositionally distinct from the original BM state.

Alpha diversity analysis further supported this ecological transition (Figure 1B). The Shannon index declined significantly in ACM (*p* = 6.623 × 10^−5^), consistent with a strong immunological bottleneck associated with colostrum production. Diversity increased again in AM, reflecting ongoing recolonization during early lactation. However, the AM microbiome did not return to the BM state, indicating that post-calving community assembly represents a lasting shift rather than a simple reversible fluctuation.

PCoA based on Bray–Curtis distances revealed a clear stage-dependent separation of microbial communities (Figure 1C). BM samples clustered tightly, reflecting relatively stable microbial exposure patterns before dry-off. ACM samples formed an even more condensed cluster, consistent with strong host-mediated ecological filtering during colostrum production. In contrast, AM samples displayed substantially increased dispersion (ANOSIM, R = 0.516, *p* = 0.001), suggesting that the microbial communities underwent individualized and heterogeneous reassembly during early lactation. This increased variability indicates that post-calving microbial succession did not follow a single deterministic trajectory but instead diverged toward multiple ecological states with greater stochasticity and inter-individual differentiation. Together, these findings support the dynamic “exposure–bottleneck–reassembly” trajectory of the mammary microbiome across the dry period.

At the phylum level, Pseudomonadota dominated all stages but fluctuated markedly in relative abundance (BM 66.76%, ACM 41.06%, AM 62.15%; Figure 1D). BM was additionally enriched in Bacteroidota and Bacillota, taxa commonly associated with environmental exposure and external microbial influx prior to teat canal closure. In contrast, ACM and AM showed increased relative abundances of Euryarchaeota, Chlamydiota, and Campylobacterota, suggesting that the post-calving microbial communities were reshaped under the combined effects of immune selection and altered nutrient availability associated with lactation.

Genus-level analysis further highlighted stage-specific ecological transitions (Figure 1E). BM communities contained higher abundances of genera that include species commonly found in environmental sources, such as *Psychrobacter*, *Denitrificimonas*, and *Thiopseudomonas*. While these patterns were consistent with increased mammary exposure before dry-off, the low-biomass nature of the milk samples warrants a cautious interpretation of these taxa. In contrast, ACM and AM communities were dominated by genera such as *Luteimonas*, *Campylobacter*, *Chlamydia*, *Methanobrevibacter*, and *Escherichia*, which include species known to respond to host-associated selective pressures. Notably, the AM microbiome remained compositionally closer to ACM than to BM, further indicating that the early lactation communities emerged from post-calving ecological filtering rather than the direct restoration of the pre-dry-off microbiota.

Overall, these results demonstrate a marked ecological restructuring of the mammary microbiome across the dry period and early lactation. The transition from the BM to ACM was characterized by a sharp reduction in microbial diversity, whereas AM showed a partial recovery but remained distinct from the pre-dry-off state, indicating persistent microbiome remodeling after calving.

#### 3.1.2. Comparative Analysis of Microbial Community Composition Across Lactation Stages

**Figure 2:**
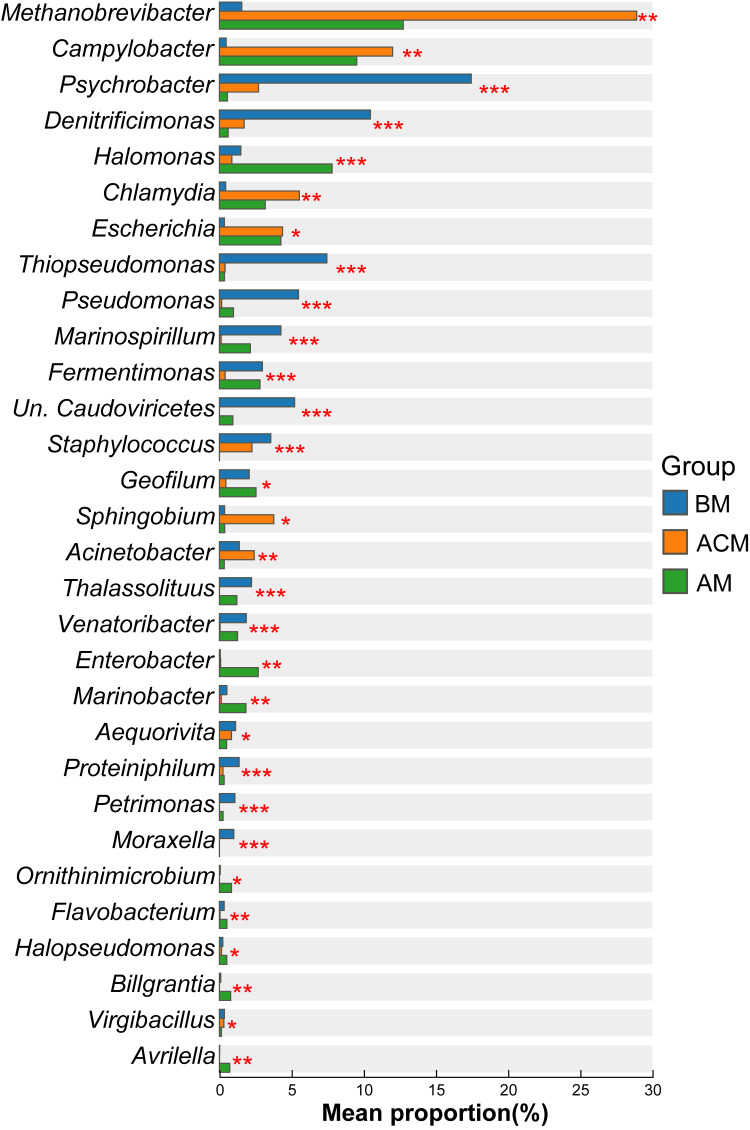
Differentially abundant genera across BM, ACM, and AM. Bar plot showing mean relative abundance (%) of genera significantly differing among the three stages. Asterisks denote significance levels: * *p* < 0.05, ** *p* < 0.01, *** *p* < 0.001.

Significant genus-level differences were observed among the BM, ACM, and AM samples, indicating marked shifts in microbial composition across lactation stages (*p* < 0.05; Figure 2). Notably, several genera previously associated with mastitis pathogenesis were significantly enriched in the BM group. Specifically, *Staphylococcus* (3.56% vs. 0.0008% in ACM and 0.00079% in AM; *p* = 0.00659), *Pseudomonas* (5.47% vs. 0.97% in ACM and 0.15% in AM; *p* = 0.00071), *Moraxella* (0.996% vs. 0% in ACM and 0.0037% in AM; *p* = 0.00030), and *Psychrobacter* (17.45% vs. 2.71% in ACM and 0.55% in AM; *p* = 0.00115) exhibited significantly higher abundances prior to dry-off. These taxa have been associated with mammary gland infection or the disruption of mucosal barrier integrity, suggesting increased susceptibility to exogenous microbial invasion during the period of incomplete teat canal closure before dry-off. Furthermore, environmentally associated genera, including *Denitrificimonas* (10.46% vs. 1.71% in ACM and 0.60% in AM; *p* = 0.00115) and *Thiopseudomonas* (7.44% vs. 0.39% in ACM and 0.34% in AM; *p* = 0.00115), were also significantly enriched in the BM group, further supporting the presence of elevated environmental microbial exposure during this stage.

In contrast, the ACM group exhibited a pronounced reduction in the abundance of potential pathogens, with no significant enrichment of canonical mastitis-associated genera. This observation is consistent with the well-established immunological characteristics of colostrum, which contains high concentrations of antimicrobial peptides, immunoglobulins, and other immune mediators that impose strong selective pressure on the mammary microbiota [29,30]. In the AM group, dominant pathogenic genera did not rebound to BM levels. Instead, several commensal or conditionally beneficial taxa, including *Bifidobacterium* (0.176% vs. 0.417% in BM and 0% in ACM; *p* = 0.01035) and *Halomonas* (7.80% vs. 1.48% in BM and 0.86% in ACM; *p* = 0.01256), showed recovery-like increases. Additionally, certain environmental genera, such as *Marinobacter* and *Venatoribacter*, displayed stage-specific enrichment, although their biological significance remains unclear. Collectively, these findings suggest a progressive ecological restructuring and partial stabilization of the mammary microbiome from colostrum through early lactation.

Overall, these data reveal a clear temporal trajectory characterized by the enrichment of potential pathogens prior to dry-off, a marked attenuation during the colostrum stage, and the absence of pathogen resurgence during early lactation. This pattern closely reflects the dynamic changes in mammary epithelial barrier function and host immune activity across the dry period, highlighting the dry-off period as a critical window of heightened susceptibility to microbial challenge.

### 3.2. ARGs Dynamics Across Lactation Stages

#### 3.2.1. Compositional and Diversity Profiles of ARGs

As dairy cows transitioned from the pre-dry-off period through calving into early lactation, a substantial restructuring of the mammary microbiome was accompanied by pronounced shifts in the composition and diversity of ARGs. Significant intergroup differences were observed among the BM, ACM, and AM samples in both ARG classes and resistance mechanisms, indicating strong stage-specific selective pressures associated with the host physiological transition.

**Figure 3:**
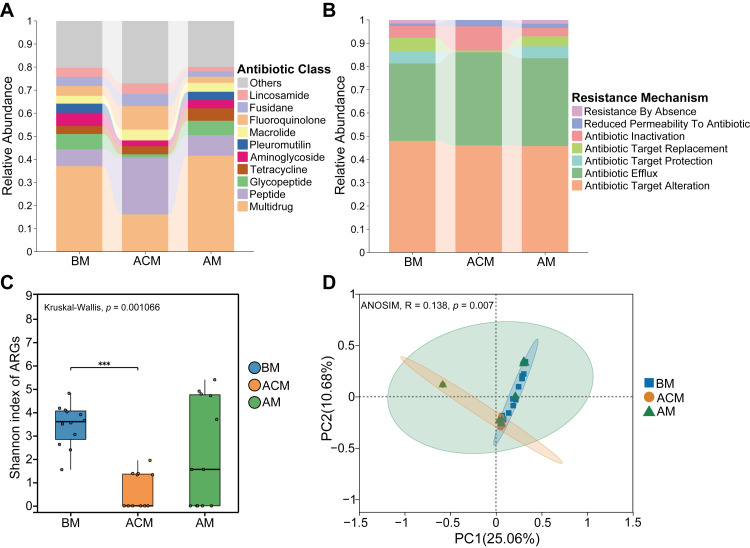
Resistome composition and diversity across BM, ACM, and AM. (**A**) Relative abundance of antibiotic resistance genes (ARG) classes across the three stages. (**B**) Relative abundance of ARG resistance mechanisms. (**C**) Shannon diversity index of ARGs. Asterisks denote significance levels: *** *p* < 0.001. (**D**) PCoA of resistome profiles. Each point represents one sample, and shaded ellipses indicate 95% confidence intervals for each group. Blue squares (BM), orange circles (ACM), and green triangles (AM) denote different sample groups.

At the antibiotic class level, multidrug, peptide, glycopeptide, and tetracycline resistance represented the dominant resistome components across all stages (Figure 3A). However, their relative abundances varied markedly over time. Multidrug resistance genes predominated in the BM group, peptide resistance genes were relatively enriched during ACM, and glycopeptide- and tetracycline-associated resistance increased progressively during AM. These shifts likely reflect dynamic ecological selection associated with dry-off and lactation recovery, including changes in teat canal permeability, immune activity, and nutrient availability within the mammary gland.

At the resistance mechanism level, antibiotic target alteration and efflux-mediated resistance consistently represented the dominant resistance strategies across all stages, suggesting that target modification and active antimicrobial extrusion constitute core adaptive mechanisms within the mammary resistome (Figure 3B). Additional mechanisms, including target protection, target replacement, and enzymatic inactivation, were detected at lower but appreciable abundances. The stage-dependent variation in these secondary mechanisms suggests ongoing functional reorganization of the resistome in response to changing ecological and host-derived selective pressures.

Alpha diversity analysis further demonstrated pronounced temporal restructuring of the resistome (Figure 3C). The Shannon diversity index of ARGs was significantly reduced in ACM compared with BM (*p* = 0.001066), consistent with a strong bottleneck effect during colostrum production. In contrast, ARG diversity partially recovered during AM, although the resistome did not fully return to the BM configuration. Beta diversity analysis similarly revealed clear stage-dependent separation of ARG profiles (PERMANOVA: R^2^ = 0.138, *p* = 0.007; Figure 3D), indicating persistent compositional divergence across lactation stages.

Collectively, these findings demonstrate that the host physiological transition across the dry period drives substantial stage-specific remodeling of the mammary resistome. The coordinated bottleneck contraction and subsequent partial recovery of ARG diversity closely paralleled microbial community succession, indicating that the ecological restructuring of the microbiome is accompanied by a synchronized functional reorganization of antimicrobial resistance potential within the mammary ecosystem.

#### 3.2.2. LEfSe-Based Identification of Stage-Specific ARG Enrichment

To further characterize stage-specific resistance signatures, a LEfSe analysis was performed at the antibiotic class level (Supplementary Table S2). Supplementary Table S2 lists all discriminative ARG categories identified by LEfSe, together with their LDA scores. All three stages exhibited significant enrichment of distinct resistance categories, supporting a strong host-driven restructuring of the mammary resistome. The BM group showed the broadest spectrum of enriched resistance classes, including lincosamide, phosphonic acid, elfamycin, fusidane, fluoroquinolone, aminoglycoside, and multidrug resistance, with high LDA scores. This expanded resistance profile likely reflects the ecologically open and environmentally exposed microbial community before dry-off. In contrast, the ACM group showed exclusive enrichment of pyrazine resistance genes. Although pyrazine antibiotics are not used in dairy cows and the absolute abundance of these genes in ACM was very low, pyrazine-related determinants are often linked to multidrug efflux systems and oxidative stress response pathways in Gram-negative bacteria. Their detection in ACM, therefore, likely reflects the survival of taxa carrying broad stress-response elements under strong colostrum-associated immune pressure, rather than true selection for pyrazine resistance. The AM group was enriched in glycopeptide and bicyclomycin-like resistance. These genes are frequently found in environmental and commensal Gram-positive bacteria and may reflect the re-expansion of taxa capable of tolerating fluctuating osmotic and nutrient conditions during early lactation. Notably, the ARG profile in AM remained distinct from BM, indicating incomplete recovery of the pre-dry-off resistome after calving.

Together, these findings demonstrate dynamic and stage-dependent restructuring of the mammary resistome across the dry-off–colostrum–early lactation continuum. The transition from BM to ACM was characterized by substantial contraction of resistance diversity, whereas AM showed partial recovery accompanied by the selective enrichment of specific ARG classes, indicating a persistent ecological remodeling of antimicrobial resistance potential within the mammary ecosystem.

### 3.3. MGEs Dynamics Across Lactation Stages

#### 3.3.1. Compositional and Diversity Profiles of MGEs

**Figure 4:**
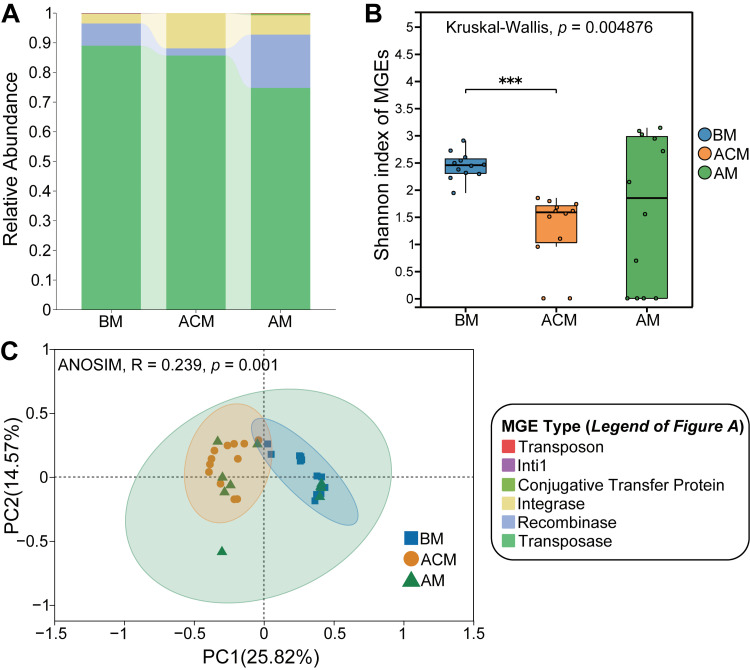
Mobilome composition and diversity across BM, ACM, and AM. (**A**) Relative abundance of major mobile genetic elements (MGE) categories. (**B**) Shannon diversity index of MGEs. Asterisks denote significance levels: *** *p* < 0.001. (**C**) PCoA of MGE profiles. Each point represents one sample, and shaded ellipses indicate 95% confidence intervals for each group. Blue squares (BM), orange circles (ACM), and green triangles (AM) denote different sample groups.

MGEs are key mediators of horizontal gene transfer and play an important role in ARG dissemination. Significant intergroup differences were observed in the relative abundances of major MGE categories, including transposases, recombinases, integrases, conjugative transfer proteins, and transposons, indicating stage-dependent shifts in genetic mobility potential (Figure 4A).

The BM group exhibited relatively high abundances of transposase- and integrase-associated elements, suggesting enhanced DNA rearrangement and horizontal gene transfer potential prior to dry-off. In contrast, the ACM group showed an enrichment of recombinase-associated MGEs, potentially reflecting increased homologous recombination activity under strong immune selection during colostrum production. During AM, transposons and several integrase-related elements became enriched, indicating a selective genomic restructuring during microbial reassembly in early lactation.

Patterns of MGE diversity closely paralleled those observed for ARGs. The ACM group exhibited the lowest Shannon diversity index (*p* = 0.004876 versus BM), consistent with immune-mediated bottlenecking during colostrum production (Figure 4B). Beta diversity analysis further revealed a significant separation among MGE profiles across stages (PERMANOVA: R^2^ = 0.239, *p* = 0.001; Figure 4C), indicating substantial restructuring of the mammary mobilome during the dry-period transition. Notably, the AM mobilome remained distinct from BM, suggesting a selective reorganization rather than the restoration of the original genetic mobility landscape after calving.

Collectively, these findings demonstrate a coordinated remodeling of the mammary mobilome across lactation stages, closely paralleling microbial and resistome succession.

#### 3.3.2. LEfSe-Based Identification of Stage-Specific MGEs Enrichment

**Figure 5:**
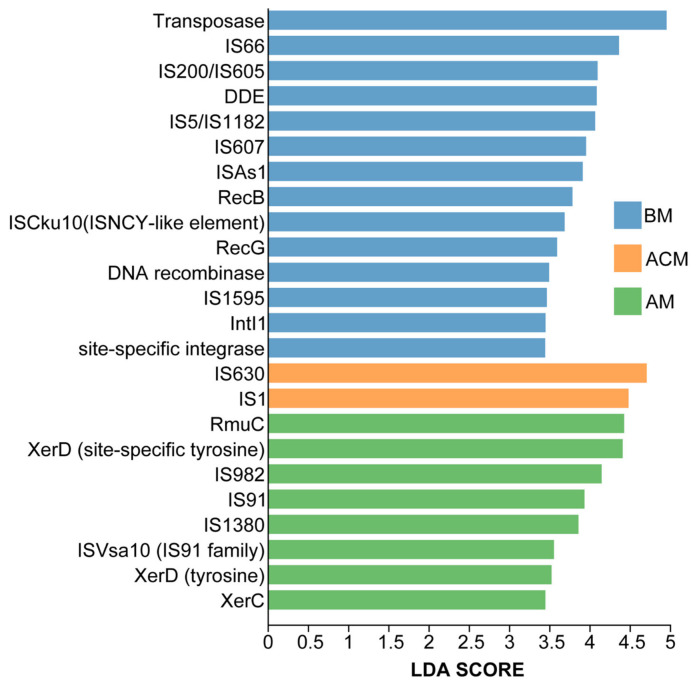
Differential enrichment of specific MGEs across lactation stages.

LEfSe analysis further resolved stage-specific MGE signatures, revealing dynamic shifts in genetic plasticity across lactation stages (Figure 5). The BM group exhibited the widest array of enriched MGEs, including IS200/IS605, DDE, IS607, ISAs1, IS5/IS1182, IS66, IS1595, ISCku10, high-abundance transposases, and diverse DNA recombination/integration enzymes (e.g., RecG, site-specific integrase). These elements are functionally linked to transposition, site-specific integration, and DNA repair, collectively signifying an elevated horizontal gene transfer potential in the pre-dry-off mammary niche. The ACM group showed the exclusive enrichment of IS630 and IS1, which are typically associated with genome reduction, stress adaptation, and transposition bursts under strong selective pressure. Their presence may reflect the survival of taxa harboring compact, mobile-element-rich genomes during the colostrum bottleneck. The AM group was uniquely enriched in IS982, RmuC, IS1380, ISVsa10, IS91, and site-specific recombinases (XerC/XerD), which are known to mediate replicative transposition, plasmid resolution, and site-specific recombination. Their increased abundance may indicate an elevated genome plasticity during microbial reassembly, potentially facilitating horizontal gene transfer or genomic adaptation as the mammary environment stabilizes. However, these interpretations remain speculative and require functional validation.

These LEfSe results align closely with the compositional and diversity trends, collectively illustrating a phased, host-orchestrated remodeling of the mammary mobilome, from maximal plasticity pre-dry-off, through a stringent restriction during colostrum, to selective re-expansion in early lactation. Such dynamics offer a crucial ecological context for understanding ARG dissemination pathways and associated risks to mammary health.

### 3.4. Co-Occurrence Network Dynamics Across Lactation Stages

**Figure 6:**
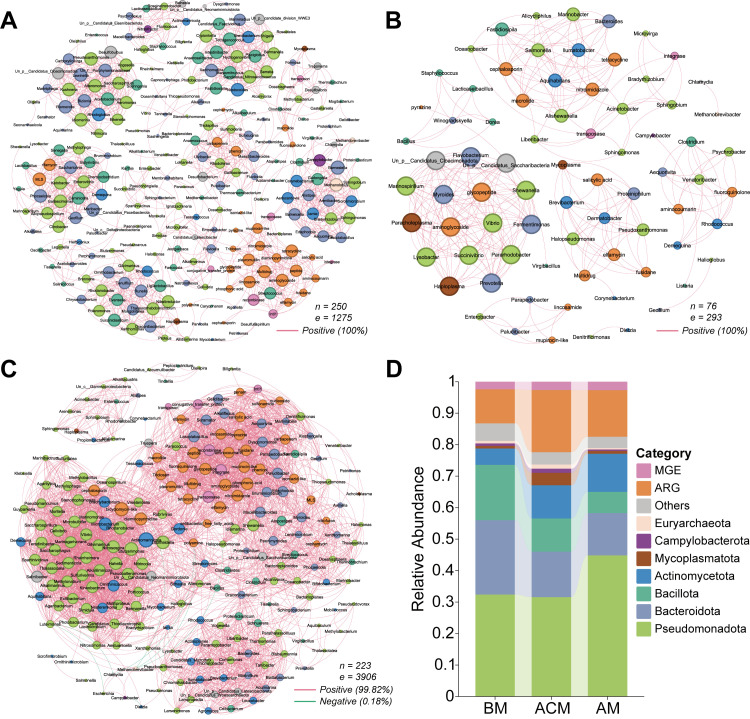
Integrated network analysis of microbial taxa, ARGs, and MGEs. (**A**) Co-occurrence network among microbial taxa, ARGs, and MGEs in BM. Average degree = 5.10, Average clustering = 0.75, Coefficient modularity = 0.88. (**B**) Co-occurrence network among microbial taxa, ARGs, and MGEs in ACM. Average degree = 3.85, Average clustering = 0.95, Coefficient modularity = 0.76. (**C**) Co-occurrence network among microbial taxa, ARGs, and MGEs in AM. Average degree = 17.51, Average clustering = 0.76, Coefficient modularity = 0.57. (**D**) Distribution of taxa and functional elements (ARGs, MGEs) contributing to network structure. Networks (12 samples per stage) were constructed using Spearman correlations based on relative abundances (genera) and TPM-normalized abundances (ARGs/MGEs). Only correlations with |r| ≥ 0.6 and FDR-adjusted *p* < 0.05 were retained. n (node) represents the number of genera, ARGs, and MGEs; e (edges) represents the number of significant correlations. Positive and negative associations are shown as red and green edges, respectively.

Co-occurrence networks constructed from the BM, ACM, and AM samples revealed marked stage-dependent differences in network structure, reflecting shifts in co-occurrence patterns across the lactation transition (Figure 6). Although these networks were correlation-based, they represent patterns of taxa and gene co-presence rather than direct ecological interactions.

The BM network exhibited the highest structural complexity and connectivity (Figure 6A). Network nodes were primarily affiliated with Pseudomonadota (32.4%) and Bacteroidota (23.6%), together with a substantial proportion of Bacillota (17.6%). Many dominant nodes belonged to environmentally associated genera, including *Marinospirillum*, *Oceanobacter*, and *Psychroflexus*, consistent with increased environmental exposure prior to dry-off. In BM, ARGs and MGEs accounted for relatively small proportions of network nodes (10.8% and 2.4%, respectively). All edges in the BM network were positively correlated, indicating that taxa tended to co-occur under similar environmental conditions, although such correlations do not imply cooperative interactions.

In contrast, the ACM network shows marked contraction and reduced connectivity (Figure 6B), consistent with strong ecological filtering during colostrum production. The relative contributions of Bacteroidota and Bacillota decreased, whereas Actinomycetota (10.53%) and Mycoplasmatota (3.95%) became more prominent. Notably, ARG-associated nodes increased to 19.74%, representing the highest proportion among the three stages, while MGE-associated nodes also increased slightly to 2.63%. These patterns suggest that resistance- and mobility-related elements are more frequently co-present with other taxa during this stage, although their increased centrality should not be interpreted as functional importance or selective advantage. Mastitis-associated genera such as *Staphylococcus*, *Chlamydia*, and *Campylobacter* remained detectable but showed reduced representation, consistent with the overall microbial bottleneck.

The AM network exhibits the greatest number of nodes (223) and edges (3906) (Figure 6C), indicating extensive co-occurrence patterns during early lactation. Pseudomonadota accounted for 44.84% of nodes, the highest proportion among the three stages, suggesting the re-establishment of dominant phyla as the mammary environment stabilized. Actinomycetota (12.11%) and Bacteroidota (13.45%) also remained important components. ARG-associated nodes decreased to 14.8% compared with ACM, whereas MGE-associated nodes increased slightly to 2.69%. Genera such as Bifidobacterium and Halomonas appeared in the AM network, consistent with progressive community diversification. Positive correlations remained predominant (99.82%), reflecting widespread co-occurrence but not necessarily cooperative ecological interactions.

Overall, the co-occurrence network analysis revealed an “exposure–bottleneck–reassembly” trajectory across lactation stages. Although these networks do not demonstrate ecological interactions or causality, their structural differences align with stage-dependent shifts in microbial composition, ARGs, and MGEs, suggesting coordinated changes in co-occurrence patterns during the dry period and early lactation.

## 4. Discussion

The mammary microbiome followed a clear “exposure–bottleneck–reassembly” trajectory across the dry period, colostrum, and early lactation. This pattern aligns with previous longitudinal studies showing pronounced ecological transitions in the mammary gland during parturition [16,31]. The open teat canal before dry-off facilitates environmental influx, while colostrum imposes strong immunological filtering, and early lactation supports gradual microbial reassembly. These stage-dependent shifts highlight the mammary gland as a dynamic ecological system shaped by host physiology and management interventions. Although genera such as *Staphylococcus*, *Pseudomonas*, and *Psychrobacter* were enriched in BM and are known mastitis-associated taxa, their presence in this stage may reflect transient environmental exposure, although low-abundance taxa in milk should be interpreted cautiously due to potential background contamination. Similar studies have shown that these genera frequently appear as low-virulence or non-pathogenic strains in dry-period milk without causing clinical mastitis, particularly when somatic cell counts remain low and no inflammatory signs are present [32].

The parallel restructuring of ARGs and MGEs indicates that functional gene pools respond to the same selective pressures driving microbial succession. The contraction of resistome and mobilome diversity during the colostrum stage, followed by a partial recovery in early lactation, mirrors patterns observed in other host-associated microbiomes under antimicrobial or immune stress [29,33,34]. The enrichment of elements such as IS91 and XerC/XerD during early lactation suggests increased genome plasticity and potential horizontal gene transfer during microbial reassembly, although the functional consequences remain to be verified experimentally.

Network analysis further supported this staged remodeling. In BM, the network was largest and most complex, while ARG and MGE nodes contributed minimally, suggesting that the structure mainly reflected the coexistence of diverse environmental taxa rather than coordinated functional interactions. In ACM, the network contracted sharply, and ARG/MGE centrality increased, consistent with strong immunological and antibiotic filtering of microbial functional traits [29,35]. In AM, the network expanded again with the reappearance of commensal-associated genera, and ARG/MGE involvement declined but remained above BM levels, indicating partial functional stabilization. Overall, these patterns highlight the transient prominence of functional genetic elements during the bottleneck phase and their gradual reduction as the community reassembles, in line with longitudinal evidence of coordinated ecological and functional remodeling across the dry period and early lactation [36,37].

Together, these findings demonstrate that the mammary microbiome undergoes coordinated ecological and functional restructuring across the dry period and early lactation. Microbial composition, ARGs, and MGEs all exhibit synchronized bottlenecks followed by a selective recovery, reflecting deep remodeling driven by host physiology and antibiotic intervention. Although early lactation communities exhibit partial ecological stabilization and a reduced abundance of several mastitis-associated taxa, the persistent divergence from the pre-dry-off microbiome, together with the incomplete recovery of ARG and MGE profiles, suggests that the mammary ecosystem reassembly after dry-off may involve long-lasting functional consequences. The AM community shows signs of compositional stabilization, yet the elevated ARGs and MGEs relative to BM indicate that the functional recovery lags behind compositional recovery, which has potential implications for udder health and antimicrobial stewardship. Whether these changes are ultimately beneficial, adaptive, or potentially detrimental to udder health requires further longitudinal investigation integrating host inflammatory responses and mastitis outcomes.

Given that milk is a low-biomass biological matrix, the possibility of background contamination cannot be fully excluded, and low-abundance environmental taxa should therefore be interpreted with caution. Furthermore, because no untreated control group was included and sampling was limited to three time points while all cows received DCT, the precise temporal sequence of microbial reassembly and the independent effects of physiology and antimicrobial exposure cannot be fully resolved. Additionally, the sample size of this study was relatively small (12 cows from a single commercial farm), which limits statistical power and constrains the generalizability of the findings to broader management systems or herd environments. To minimize intra-animal variability, only one quarter per cow was sampled, but this design does not capture potential quarter-level heterogeneity in microbial or resistome dynamics. Future work integrating metagenome-assembled genomes, host immune profiling, and longitudinal sampling will be essential to identify the key drivers of microbial reassembly and to evaluate how different dry-off strategies influence the long-term resistome and mobilome of the mammary gland.

## Data Availability

The original data presented in this study are openly available in NCBI under the project number PRJNA1455201 at https://www.ncbi.nlm.nih.gov/ (accessed on 3 June 2026).

## Supplementary Materials

The following supporting information can be downloaded at: https://www.mdpi.com/article/10.3390/vetsci13060559/s1, Table S1: Summary of assembly statistics and gene annotation for metagenomic datasets. Table S2: LEfSe analysis of ARGs classes across BM, ACM, and AM.

## Abbreviations

IMI	Intramammary Infection
DCT	Dry Cow Therapy
ARGs	Antibiotic Resistance Genes
SCC	Somatic Cell Count
ORFs	Open Reading Frames
COG	Clusters of Orthologous Groups
MGEs	Mobile Genetic Elements
FDR	False Discovery Rate
PCoA	Principal Coordinate Analysis
LEfSe	Linear Discriminant Analysis Effect Size
